# Effect of *Lactiplantibacillus plantarum* Fermentation on the Structural and Functional Properties of Rice Protein/Carboxymethylcellulose Stabilized High-Internal-Phase Emulsion

**DOI:** 10.3390/foods15213995

**Published:** 2026-11-07

**Authors:** Yurong Zhou, Jing Yang, Wenfei Xiong, Chao Chen, Lifeng Wang

**Affiliations:** 1 College of Food Engineering, Harbin University of Commerce, Harbin 150028, China; 2 College of Food Science and Engineering, Nanjing University of Finance and Economics, Nanjing 210023, China

**Keywords:** rice protein, CMC, fermentation, high-internal-phase emulsions, spreadable sauces

## Abstract

Plant-based high-internal-phase emulsions (HIPEs) are promising structured fat systems for spreadable sauces, but their performance depends on stabilizer composition and processing. In this study, oil-in-water HIPEs (oil-phase volume fraction 0.75) were prepared using rice protein/carboxymethyl cellulose (RP/CMC) complexes at mass ratios of 10/0–10/4, with or without *Lactiplantibacillus plantarum* fermentation. Oil-binding capacity (OBC), rheological properties, response to heating (90 °C for 60 min), and microstructure were evaluated. CMC incorporation improved self-supporting behavior, macroscopic homogeneity, and OBC compared with RP alone. The 10/3 formulation showed the numerically highest OBC (99.84 ± 0.13% for unfermented and 99.69 ± 0.04% for fermented samples), with no significant difference from the corresponding 10/4 formulation. All HIPEs exhibited shear-thinning and elastic-dominant, gel-like behavior. Fermentation reduced apparent viscosity and storage modulus, indicating a less rigid and more deformable structure. After heating, samples maintained their semi-solid appearance and oil-in-water dispersion characteristics. Microscopy showed more compact droplet arrangements in CMC-containing systems, whereas fermentation was associated with a less compact apparent arrangement. These findings indicate that RP/CMC complexes can structure plant-based HIPEs and that fermentation can modulate their rheological properties; storage stability and direct spreadability require further evaluation.

## 1. Introduction

With increasing consumer attention to healthy diets, sustainable foods, and the replacement of animal-derived ingredients, plant-based foods have become an important direction in food product development [1]. High internal phase emulsions (HIPEs) are a highly promising class of structured fat systems, suitable for use in plant-based mayonnaise-type products, due to their ability to form semi-solid or gel-like network structures through the tight packing of droplets, as well as their tunable rheological properties and exceptional oil-holding capacity [2,3]. Plant-protein-stabilized HIPEs based on wheat gluten, yellow mustard seed protein, soy protein, and pea protein have been investigated as potential alternatives to conventional mayonnaise-like products [4,5,6,7].

Among various plant proteins, rice protein (RP) has attracted increasing attention due to its wide availability, high nutritional value, low allergenicity, and good consumer acceptance [8]. However, RP tends to aggregate in aqueous systems, resulting in limited water dispersibility and interfacial activity, which restricts its application in emulsion-based foods [9]. Previous studies have mainly improved the solubility and techno-functional properties of rice protein through enzymatic hydrolysis, ultrasonic treatment, or pH cycling. However, these treatments may induce excessive structural changes in proteins, loss of nutritional components, or the formation of undesirable flavors, thereby affecting their applicability in food systems [10,11,12]. In contrast, protein–polysaccharide complexation is a relatively mild structural modulation strategy. Rice protein can form complexes with polysaccharides through non-covalent interactions such as electrostatic interactions, hydrogen bonding, and hydrophobic interactions [13]. Studies have shown that during complex formation, rice protein undergoes conformational rearrangement and interfacial behavior modulation, thereby improving functional properties such as emulsifying and foaming abilities [14]. Carboxymethyl cellulose (CMC) is a commonly used anionic polysaccharide with good thickening, stabilizing, and structure-modulating properties [15]. Previous studies have shown that regulating the electrostatic interaction between RP and CMC can significantly improve the water dispersibility and functional properties of rice protein [14]. Therefore, using RP/CMC complexes to stabilize HIPEs is expected to combine the interfacial adsorption ability of protein with the continuous-phase thickening effect of polysaccharide, thereby constructing plant-based high-oil sauce systems with good oil retention and semi-solid texture.

In addition to protein–polysaccharide complexation, microbial fermentation is also an important strategy for improving the quality of plant protein-based foods. Lactic acid bacterial fermentation can alter the molecular structure, aggregation state, and functional properties of plant proteins through acidification, hydrolysis, or metabolic transformation, thereby affecting their processing properties such as solubility, emulsifying capacity, and gelation ability [16]. Meanwhile, fermentation may reduce undesirable off-flavors in plant proteins and generate organic acids, peptides, and volatile flavor compounds, potentially modifying the flavor-related properties of plant-based foods [17]. Previous studies have shown that *Lactiplantibacillus plantarum* fermentation can improve the stability and sensory characteristics of polysaccharide-stabilized rice protein beverages [18]. In addition, studies on fermentation-induced plant protein emulsion gels have shown that different lactic acid bacterial combinations can improve the textural properties of pea protein emulsion gels and affect their viscoelasticity and mechanical strength by regulating the protein matrix and oil droplet network structure [19]. These findings suggest that fermentation may serve as a strategy for regulating the functional properties, network structure, and rheological behavior of plant protein-based emulsion systems.

In RP/CMC-HIPEs systems, the effect of fermentation may not only originate from changes in the structure of rice protein itself, but may also be closely related to changes in the interactions between RP and CMC. Protein–polysaccharide interactions are central to the construction of stable emulsion systems. The non-covalent interactions formed between proteins and polysaccharides can regulate the solubility, interfacial adsorption behavior, continuous-phase viscosity, and network structure of the complexes, thereby affecting the formation, stability, and rheological properties of HIPEs [20]. Recent studies on HIPEs stabilized by rice protein–xanthan gum complexes have shown that non-covalent interactions can improve the solubility, interfacial properties, and viscoelasticity of rice protein, and promote the formation of a stable droplet-packing network in HIPEs [21]. However, the structural properties of protein–polysaccharide complexes are highly sensitive to pH, protein conformation, and processing conditions. Fermentation can induce changes in system pH, partial protein hydrolysis, or rearrangement of protein aggregation states. These changes may further influence the interfacial adsorption behavior and continuous-phase structure of RP/CMC complexes, ultimately altering the oil-holding capacity, viscoelasticity, thermal stability, and spreadability of HIPEs [22,23]. At present, research on how fermentation regulates protein–polysaccharide interactions and further affects the structural stability and sauce applicability of plant-based HIPEs remains limited.

Therefore, in this study, unfermented and fermented RP/CMC complexes were used as the continuous phase, and soybean oil was used as the dispersed phase to prepare O/W HIPEs with an oil phase volume fraction of 0.75 through a one-step shear homogenization method. The effects of RP/CMC mass ratio and fermentation treatment on the macroscopic appearance, dispersion behavior, centrifugal stability, rheological properties, thermal stability, and microstructure of HIPEs were systematically investigated. This study aimed to determine how fermentation modulates the rheological properties and microstructure of RP/CMC-HIPEs and whether high oil-binding capacity and semi-solid structure can be retained after the tested thermal treatment. The potential relevance of the fermentation-induced rheological changes to spreadable-sauce applications was also considered. The results are expected to provide insights into the structural and functional changes associated with fermentation in RP/CMC-stabilized HIPEs and provide a theoretical basis for developing plant-based mayonnaise-like sauce alternatives.

## 2. Materials and Methods

### 2.1. Materials

Rice protein powder containing 91.2 wt% rice protein was purchased by Anhui Shunxin Shengyuan Biological Food Co., Ltd. (Chuzhou, China). CMC (Degree of substitution of 1.2, molecular weight of 250 kDa), Nile red and Nile blue A were purchased by Merck KGaA (Darmstadt, Germany). Soybean oil and white granulated sugar were purchased by Yihai Kerry Arawana Holdings Co., Ltd. (Shanghai, China). *Lactiplantibacillus plantarum* CGMCC 1.2469 was obtained from China General Microbial Strain Preservation Center (Beijing, China). All other reagents were of analytical grade.

### 2.2. Preparation of Fermented RP/CMC Complexes

The preparation and fermentation of the RP/CMC complex were carried out according to the previously optimized method [18]. Rice protein (RP) powder was dissolved in 0.04 mol/L NaOH, and the supernatant was collected to obtain a 10 mg/mL protein solution. CMC was added to give RP/CMC mass ratios of 10/0, 10/1, 10/2, 10/3, 10/4. After thorough mixing, the pH was adjusted to 5.2 using a 0.1 mol/L HCl, 3.5% (*w*/*v*) sucrose was added, and the mixture was blended for 30 min using a vortex mixer (DMT-2500, Hangzhou Meio Instrument Co., Ltd., Hangzhou, China) and subsequently homogenized twice at a pressure of 30 MPa (AMH-MINI, Antos Nano Technology (Suzhou) Co., Ltd., Suzhou, China). The dispersion was pasteurized at 90 ± 2 °C for 30 min and then cooled to 37 °C. *Lactiplantibacillus plantarum* was inoculated into sterile MRS medium broth for activation and incubated at 37 °C for 12 h until the viable cell concentration reached 1 × 10^8^ CFU/mL. The activated bacterial suspension was then inoculated into the cooled RP/CMC complex at 2.0% (*v*/*v*) and incubated at 37 °C for 3 h. After fermentation, both the unfermented samples and the fermented samples were subjected to heat treatment at 90 ± 2 °C for 10 min, and the resulting RP/CMC complexes were cooled to 4 °C for subsequent use.

### 2.3. Preparation of RP/CMC-HIPEs

The fermented and unfermented RP/CMC complexes were used as the continuous phase and soybean oil as the dispersed phase. The continuous phase and dispersed phase were mixed at a volume ratio of 1:3 (*v*/*v*), with the final oil-phase volume fraction (*φ*) adjusted to 0.75. The mixture was emulsified with a high-shear homogenizer (IKA T50, IKA Corp., Staufen, Baden-Württemberg, Germany) at 8000 rpm for 1 min to obtain oil-in-water high-internal-phase emulsions (O/W HIPEs).

### 2.4. Dispersion of the RP/CMC-HIPEs

Based on the method of Yang et al. [24] with minor modifications, the dispersion of the RP/CMC-HIPEs system was determined. Freshly prepared RP/CMC-HIPEs (0.20 g) were transferred into centrifuge tubes and separately dispersed in distilled water and soybean oil at a mass ratio of 1:9, respectively. After standing for 12 h at room temperature, the samples were vortexed for 30 s and their dispersion behavior was analyzed.

### 2.5. Centrifugal Stability and Oil Binding Capacity of the RP/CMC-HIPEs

The centrifugal stability of RP/CMC-HIPEs was evaluated using an accelerated centrifugation method [25]. In total, 10 g of samples ($W_{i}$) was transferred into centrifuge tubes and centrifuged at 10 °C and 10,000× *g* for 30 min. After centrifugation, oil release and phase separation were visually recorded. The released free oil was removed using a pipette, and the mass of the remaining phase ($W_{f}$) was measured. The oil-binding capacity (OBC) was calculated according to Equation (1).

(1) $$OBC\left(\%\right)=\left(1-\frac{Wi-Wf}{Wi}\right)\times 100$$

where $W_{i}$ represents the mass (g) of initial sample before centrifugation, and $W_{f}$ represents the mass (g) of the remaining phase after centrifugation and removal of the released free oil.

### 2.6. Thermal Stability of the RP/CMC-HIPEs System

Fresh RP/CMC-HIPEs were sealed in sample bottles, heated in a 90 °C water bath for 60 min and cooled to room temperature [6]. The macroscopic appearance, microstructure and rheological properties before and after heating were compared with an unheated control. The viscoelastic property was measured using a rheometer.

#### 2.6.1. Rheological Properties of the RP/CMC-HIPEs

Rheology was measured with a rotational rheometer (MCR302, Anton Paar Corporation, Graz, Austria) using a PP50 parallel-plate measuring system with a gap of 1 mm at 25 °C (test temperature) [26]. After loading, the samples were equilibrated at the test temperature for 1 min prior to measurement. Steady shear measurements were carried out over a shear rate range of 0.1–100 s^−1^. Dynamic oscillatory measurements included strain sweep and frequency sweep tests. The strain sweep was performed at an angular frequency of 6.283 rad/s over a strain range of 0.1–100%. The frequency sweep was conducted at a constant strain of 0.1% over a frequency range of 0.1–100 rad/s to determine the storage modulus (G′) and loss modulus (G″). Rheological measurements were conducted using three independently prepared HIPE samples (*n* = 3), with each sample measured under the same experimental conditions.

#### 2.6.2. Microstructural Characterization of the RP/CMC-HIPEs

The microstructure of RP/CMC-HIPEs was observed using confocal laser scanning microscopy (CLSM) and cryo-scanning electron microscopy (Cryo-SEM).

The microscopic structure of RP/CMC-HIPEs was observed using a confocal laser scanning microscope (Leica TCS SPE, Leica Microsystems, Heidelberg, Germany) [27]. RP/CMC-HIPEs were respectively stained with the fluorescent dyes Nile Red and Nile Blue A for the oil phase and protein phase. An appropriate amount of HIPEs was placed in a centrifuge tube, and 0.1% (*w*/*v*) Nile Red and 0.1% (*w*/*v*) Nile Blue A staining solutions were mixed at a volume ratio of 1:1. The HIPEs were then stained in the dark for 30 min. Images were collected under excitation wavelengths of 488 nm and 635 nm.

The microstructure of the RP/CMC-HIPEs was examined using a field-emission scanning electron microscope equipped with a cryo-transfer system (XL30FEG Cryo-SEM, Royal Philips of the Netherlands, Eindhoven, the Netherlands). Fix the RP/CMC-HIPEs onto the sample stage and quickly freeze them by immersing them in liquid nitrogen. After the freezing fixation, transfer the samples to the low-temperature preparation room (PP3000T Cryo Transfer System, Quorum Technologies LTD, Lewes, East Sussex, UK) for sublimation etching treatment. Subsequently, apply platinum spraying treatment with a current of 10 mA for 90 s to the RP/CMC-HIPEs. Finally, observe the microscopic structure of the frozen section of the sample under an accelerating voltage of 5 kV [28].

### 2.7. Statistical Analysis

Quantitative data are presented as mean ± SD. Oil-binding capacity (OBC) was analyzed using two-way analysis of variance (ANOVA), with RP/CMC ratio and fermentation treatment as fixed factors, including their interaction. When a significant interaction was detected, simple-effects analyses were performed with appropriate adjustments for multiple comparisons. The functional properties of unfermented and fermented RP/CMC complexes presented in Table S1 were analyzed separately using one-way ANOVA, with fermentation treatment as the single factor. Statistical significance was set at *p* < 0.05. Statistical analyses were performed using IBM SPSS Statistics 22.0, and figures were prepared using OriginLab Origin 2018.

## 3. Results and Discussion

### 3.1. The Macroscopic Morphology and Dispersion Characteristics of RP/CMC-HIPEs

**Figure 1:**
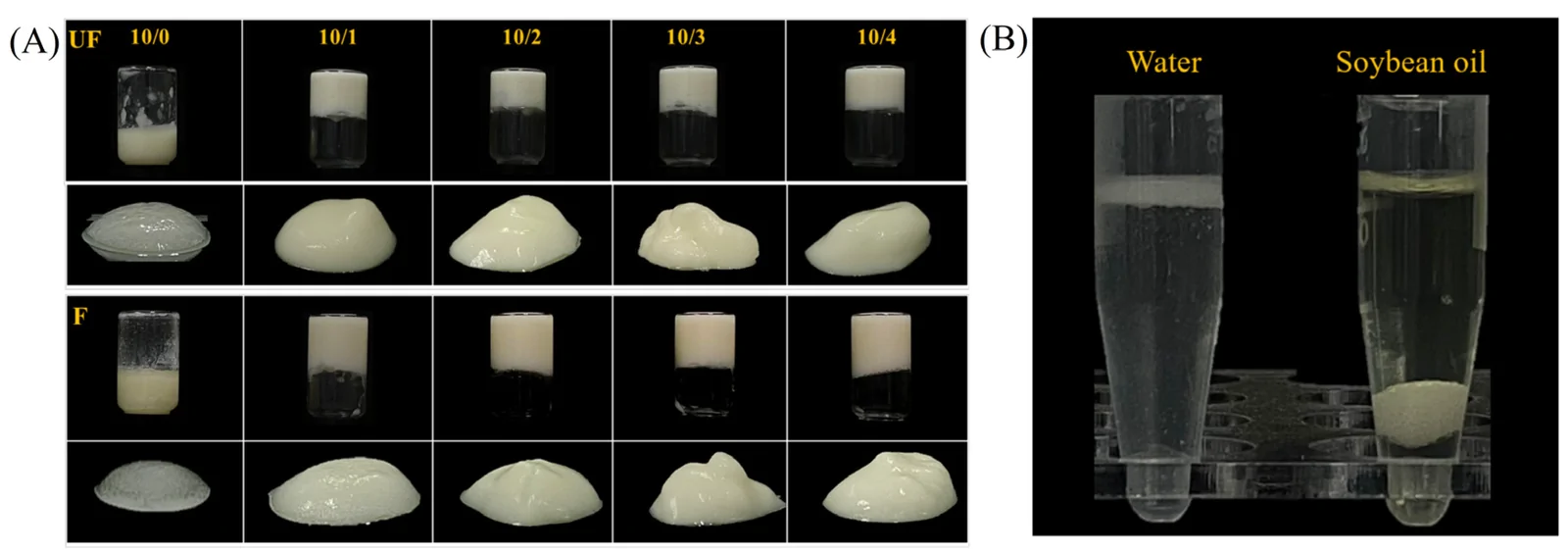
(**A**) The macroscopic appearance of RP/CMC-HIPEs and (**B**) F-10/3 sample dispersion behavior in water and soybean oil. UF represents the unfermented sample and F represents the fermented sample, as in the following cases.

Figure 1 presents the macroscopic appearance and dispersibility of HIPEs stabilized by RP/CMC complexes with different mass ratios. Using RP/CMC complexes as the continuous phase and soybean oil as the dispersed phase, HIPEs with an oil volume fraction (φ) of 0.75 were successfully prepared through a one-step high-shear homogenization process. As shown in Figure 1B, the prepared HIPEs could be uniformly dispersed in deionized water but not in soybean oil, confirming that the emulsions were oil-in-water (O/W) HIPEs.

As shown in Figure 1A, the HIPEs stabilized solely by rice protein (10/0) exhibited poor self-supporting ability and a heterogeneous appearance. With increasing CMC content from 10/1 to 10/3, the samples became more homogeneous and developed a semi-solid, sauce-like appearance, remaining stationary when the containers were inverted. Further increasing the CMC content to a ratio of 10/4 produced no obvious additional improvement in macroscopic appearance. At the same RP/CMC ratio, the fermented samples retained their overall semi-solid structure and homogeneity, although they appeared visually softer than the corresponding unfermented samples. This is because lactic acid fermentation may alter the conformation, aggregation behavior, and functional properties of rice proteins, potentially affecting the organization of the resulting RP/CMC-HIPEs [29]. These observations indicate that CMC incorporation was associated with improved macroscopic uniformity and self-supporting behavior, whereas fermentation altered the apparent firmness of the HIPEs without causing visible macroscopic collapse.

### 3.2. Oil-Binding Capacity of RP/CMC-HIPEs

**Figure 2:**
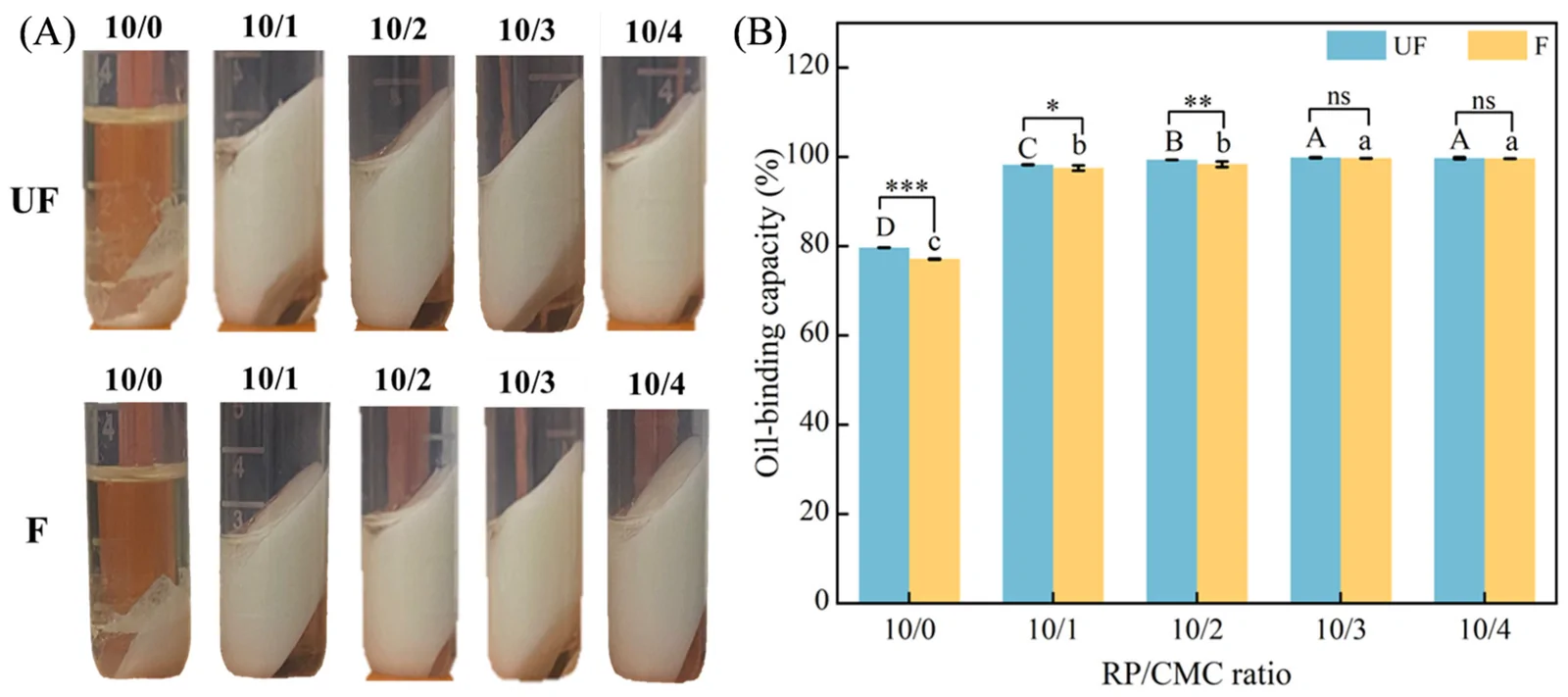
(**A**) Macroscopic appearance of RP/CMC-HIPEs after centrifugation at 10 °C and 10,000× *g* for 30 min and (**B**) oil-binding capacity of UF and F samples. * Different uppercase letters indicate significant differences among RP/CMC ratios within the UF treatment, while different lowercase letters indicate significant differences among RP/CMC ratios within the F treatment. Asterisks indicate significant differences between UF and F at the same RP/CMC ratio (* *p* < 0.05, ** *p* < 0.01, *** *p* < 0.001; ns, not significant).

Since RP/CMC-HIPEs exhibited semi-solid characteristics, accelerated centrifugation was employed as a complementary method to compare their resistance to oil release across different formulations [6]. As shown in Figure 2A, obvious phase separation was observed in both unfermented and fermented 10/0 samples after centrifugation. This indicates that the interfacial layer and continuous-phase network formed by RP alone were insufficient to immobilize the highly concentrated oil phase. In contrast, the RP/CMC composite systems (10/1–10/4) exhibited only slight surface oil separation after centrifugation, indicating markedly improved stability. The numerically highest oil-binding capacities were observed at an RP/CMC ratio of 10/3, reaching 99.84 ± 0.13% and 99.69 ± 0.04% for the unfermented and fermented samples, respectively. However, these values were not significantly different from those obtained at a ratio of 10/4. These results indicate that CMC markedly enhanced the ability of HIPEs to retain the oil phase and resist oil droplet migration and phase separation during centrifugation, consistent with the improved macroscopic uniformity observed in Figure 1 [30].

In addition, the effect of fermentation on HIPE stability was closely associated with the CMC content. OBC decreased after fermentation in the 10/0, 10/1, and 10/2 systems, whereas the 10/3 and 10/4 formulations retained high OBC values without significant differences between the unfermented and fermented samples. This decrease may be associated with fermentation-induced changes in rice protein structure and functionality. Proteolytic activity associated with *Lactiplantibacillus plantarum* may alter protein molecular characteristics and conformation, thereby affecting techno-functional properties such as solubility, emulsifying capacity and gelation behavior [16]. Previous studies have also shown that proteolytic systems from lactic acid bacteria can modify legume proteins and modulate their functional properties [22].

### 3.3. Rheological Properties of RP/CMC-HIPEs

**Figure 3:**
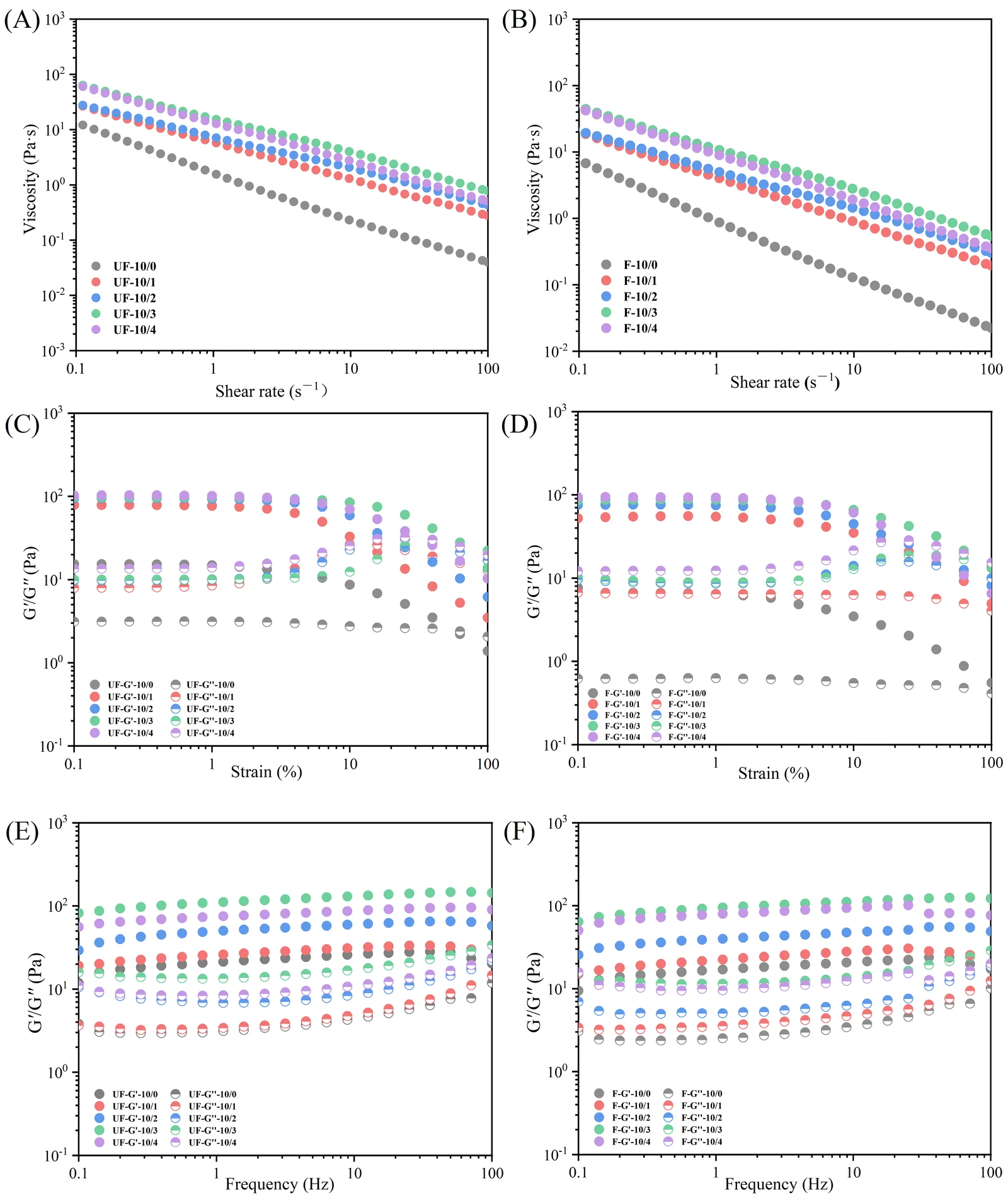
Apparent viscosity curves (**A**,**B**), strain sweep curves (**C**,**D**), and frequency sweep curves (**E**,**F**) of unfermented (UF) and fermented (F) RP/CMC-HIPEs.

Figure 3 presents the steady-state rheological behavior and dynamic viscoelastic properties of unfermented and fermented RP/CMC-HIPEs. As shown in Figure 3A,B, the apparent viscosity of all samples decreased with increasing shear rate, exhibiting typical shear-thinning behavior, characteristic of non-Newtonian fluids. This phenomenon may reflect the partial disruption or rearrangement of droplet associations and the continuous-phase structure under high shear rates, resulting in reduced apparent viscosity. Such rheological behavior is advantageous for spreadable sauce products because it allows the system to maintain its shape at rest while exhibiting good flowability during extrusion, spreading, and oral processing. Previous studies have reported that mayonnaise-like emulsions typically exhibit shear-thinning behavior and weak-gel characteristics, and that their rheological properties are closely related to product stability and consumer acceptance [26,31]. With increasing CMC content up to an RP/CMC mass ratio of 10/3, the apparent viscosity increased progressively, whereas no further increase was observed at a ratio of 10/4. This result suggests that the increased flow resistance with CMC addition may be associated with continuous-phase thickening and possible interactions between RP and CMC, which may also contribute to oil retention. Compared with the corresponding unfermented samples, fermented RP/CMC-HIPEs exhibited lower apparent viscosity at the same RP/CMC mass ratio, suggesting that fermentation reduced the structural resistance of the RP/CMC-HIPE system, thereby facilitating deformation and flow. Such rheological changes may favor easier deformation during spreading.

Figure 3C,D show that the storage modulus (G′) remained higher than the loss modulus (G″) within the low-strain region, indicating that RP/CMC-HIPEs possessed an elastic-dominant gel-like structure [32]. G′ increased as the RP/CMC ratio changed from 10/0 to 10/3, whereas no substantial additional increase was observed at 10/4. With increasing strain, a decrease in G′ and a crossover between G′ and G″ were observed, indicating progressive disruption of the gel network as the applied strain exceeded the linear viscoelastic region [23,33]. In the fermented samples, G′ remained higher than G″ but was lower than that of the corresponding unfermented samples, indicating a reduced elastic response while preserving the gel-like rheological behavior. During frequency sweeps (Figure 3E,F), G′ exhibited weak frequency dependence and remained higher than G″, further supporting the weak-gel characteristics of the systems. Similar fermentation-related changes in viscoelastic properties have been reported for pea-protein emulsion gels [19].

As shown in Figure 3E,F, both unfermented and fermented RP/CMC-HIPEs exhibited weak frequency dependence of G′ over the tested frequency range, further confirming the weak-gel characteristics of the systems. Compared with the corresponding unfermented samples, the fermented RP/CMC-HIPEs maintained G′ > G″ while exhibiting lower G′ values, indicating that fermentation reduced the rigidity of the RP/CMC-HIPE system without disrupting its gel-like network structure. This observation is consistent with the lower apparent viscosity of the fermented samples shown in Figure 3A,B. Similar observations have been reported for pea protein emulsion gels, where fermentation with different combinations of lactic acid bacteria modified the viscoelastic and mechanical properties of the systems by altering the organization of the protein matrix and droplet network [19]. These findings suggest that fermentation reduced the elastic response of the emulsion system while preserving its gel-like rheological behavior. For spreadable sauce products, a lower G′ may indicate reduced elastic resistance to deformation, which could be favorable for spreading-related handling.

### 3.4. CLSM Images and Particle Size Analysis of RP/CMC-HIPEs

**Figure 4:**
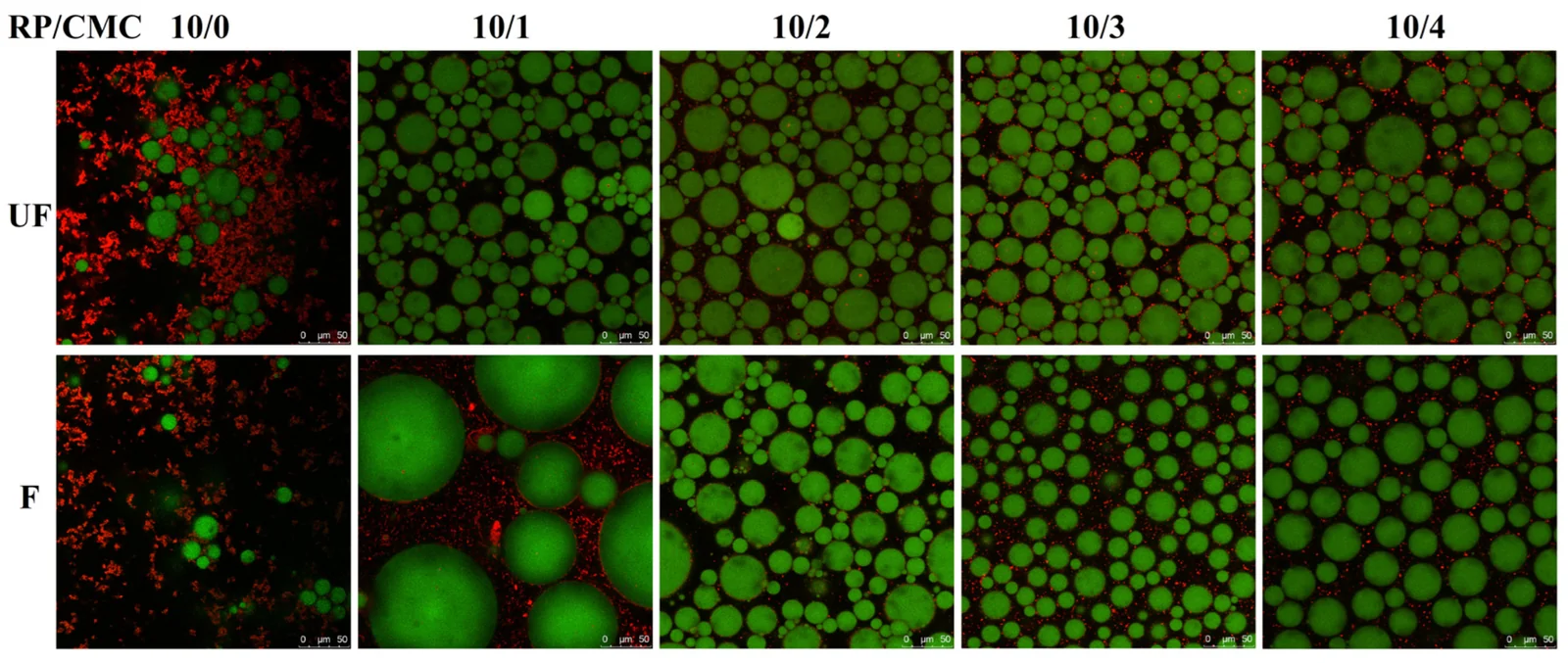
CLSM images of RP/CMC-HIPEs before and after fermentation.

**Figure 5:**
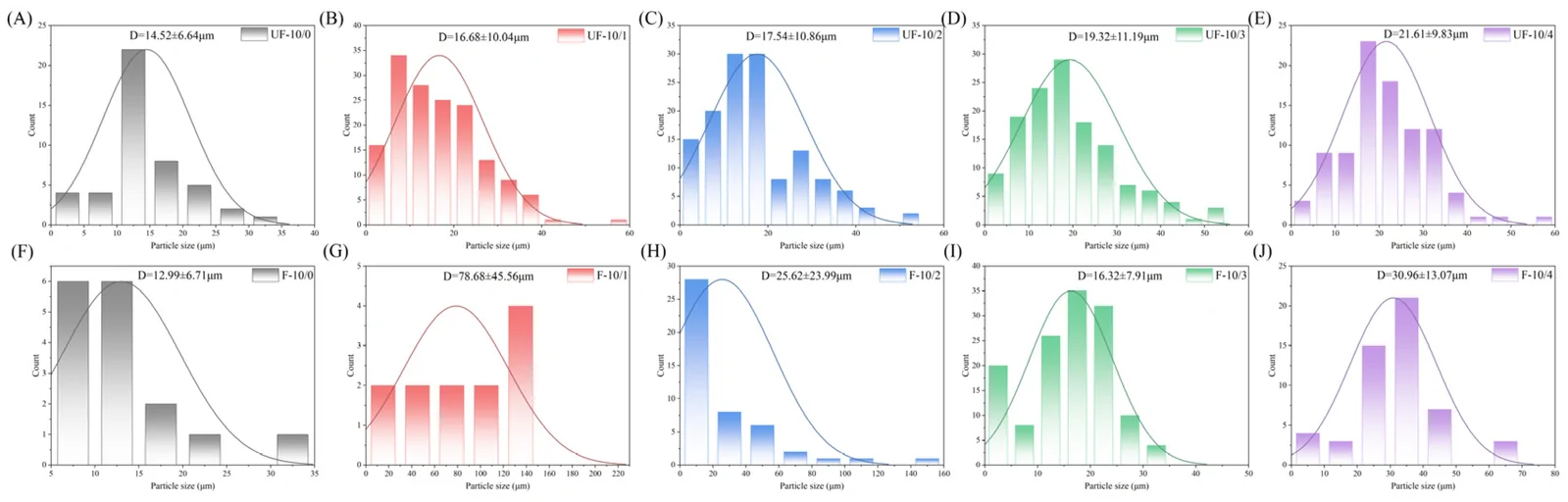
The particle size distribution of RP/CMC-HIPEs: (**A**–**E**) are the samples before fermentation, and (**F**–**J**) are the samples after fermentation.

The CLSM images of RP/CMC-HIPEs are shown in Figure 4, and the particle size analysis results are shown in Figure 5. The oil phase stained with Nile Red is shown in green, while rice protein stained with Nile Blue appears in red. All RP/CMC-HIPEs exhibited a close-packed droplet structure, indicating the successful formation of typical high-internal-phase emulsions at an oil volume fraction of *φ* = 0.75 [34]. In the absence of CMC, the 10/0 sample exhibited a heterogeneous droplet distribution with obvious droplet aggregation, suggesting that RP alone may provide less effective droplet stabilization under high oil-loading conditions. The incorporation of CMC markedly improved the microstructure of the HIPEs. In the 10/1–10/4 samples, the oil droplets were more uniformly distributed, and the red protein fluorescence was mainly localized around the oil droplets, suggesting that RP participated in the association of RP with the droplet boundaries. The interaction between RP and CMC may contribute to a more uniform droplet arrangement, with their higher apparent viscosity and oil-binding capacity, potentially through changes in continuous-phase viscosity, interfacial organization, or both [35]. In particular, both the unfermented and fermented samples with an RP/CMC mass ratio of 10/3 exhibited a relatively compact and homogeneous droplet arrangement, suggesting that an appropriate amount of CMC effectively improved the microstructure of RP-stabilized HIPEs. Furthermore, the fermented RP/CMC-HIPEs maintained a continuous and closely packed droplet network, indicating that fermentation did not compromise the microstructural integrity of the HIPEs. Compared with the corresponding unfermented samples, the droplet packing in the fermented systems appeared slightly less compact while the protein phase remained closely associated with the oil droplets. This observation is consistent with the rheological measurements: the fermented RP/CMC-HIPEs exhibit lower apparent viscosity and storage modulus (G′), indicating that fermentation leads to a looser packing of the droplets and a reduction in structural compactness, though no significant, large-scale structural breakdown was observed.

### 3.5. The Macroscopic Morphology and Dispersion Characteristics of the Heated RP/CMC-HIPEs

**Figure 6:**
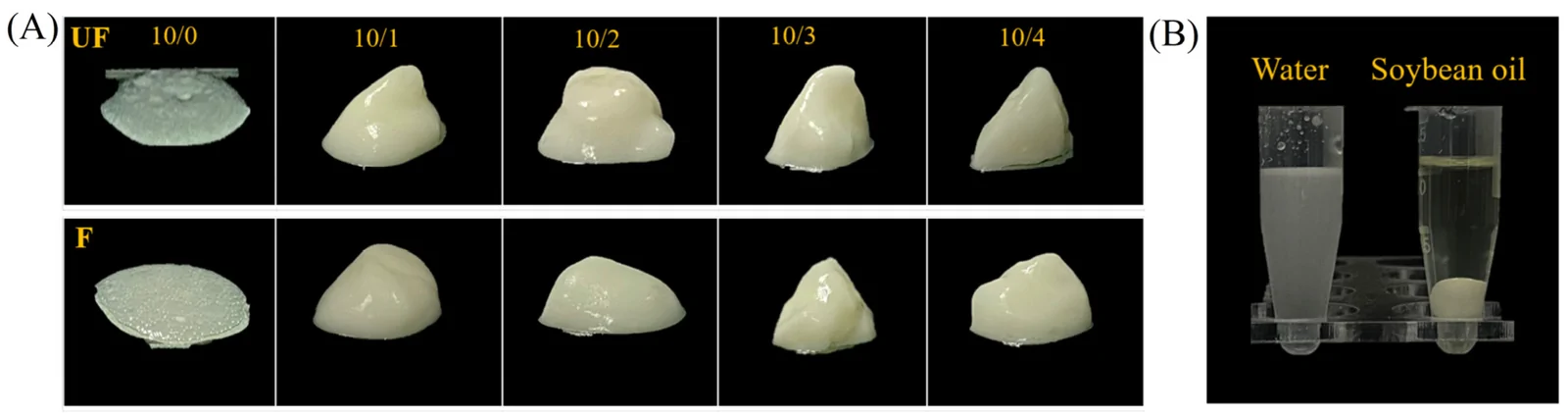
(**A**)The macroscopic appearance of RP/CMC-HIPEs after heating at 90 °C for 60 min and (**B**) F-10/3 sample dispersion behavior in water and soybean oil.

Figure 6 shows the macroscopic appearance and dispersion behavior of RP/CMC-stabilized HIPEs after heating at 90 °C for 60 min. Thermal treatment is a common sterilization and processing operation in the manufacture of sauce products; therefore, the ability of HIPEs to maintain their structure under elevated temperatures is closely related to their suitability for practical food processing applications. As shown in Figure 6A, all samples retained their semi-solid, sauce-like appearance after heating, and no obvious oil release or structural collapse was observed, indicating that RP/CMC-stabilized HIPEs possessed good thermal stability. Compared with the 10/0 sample, the RP/CMC composite systems exhibited better shape retention, suggesting that interactions between RP and CMC may have contributed to maintaining the overall structure of the HIPEs during thermal treatment [36].

As shown in Figure 6B, the heated RP/CMC-HIPEs formed a homogeneous milky-white suspension in the aqueous phase but remained undispersed in soybean oil, indicating that thermal treatment did not alter their oil-in-water (O/W) emulsion type. Compared with the dispersion behavior before heating (Figure 1B), the samples changed from a relatively clear dispersion to a milky-white suspended system after heating, suggesting that thermal treatment modified the colloidal dispersion behavior of HIPEs in water. This change may reflect heat-induced changes in the apparent size or organization of dispersed structures, which could alter the light-scattering behavior of the system [24]. Nevertheless, these changes did not result in emulsion destabilization, as evidenced by the absence of obvious oil release and the preservation of the semi-solid structure. Furthermore, the fermented samples also retained their semi-solid appearance and O/W dispersion characteristics after heating, indicating that fermentation did not compromise the thermal stability of RP/CMC-HIPEs.

### 3.6. The Rheological Properties of Heated RP/CMC-HIPEs

**Figure 7:**
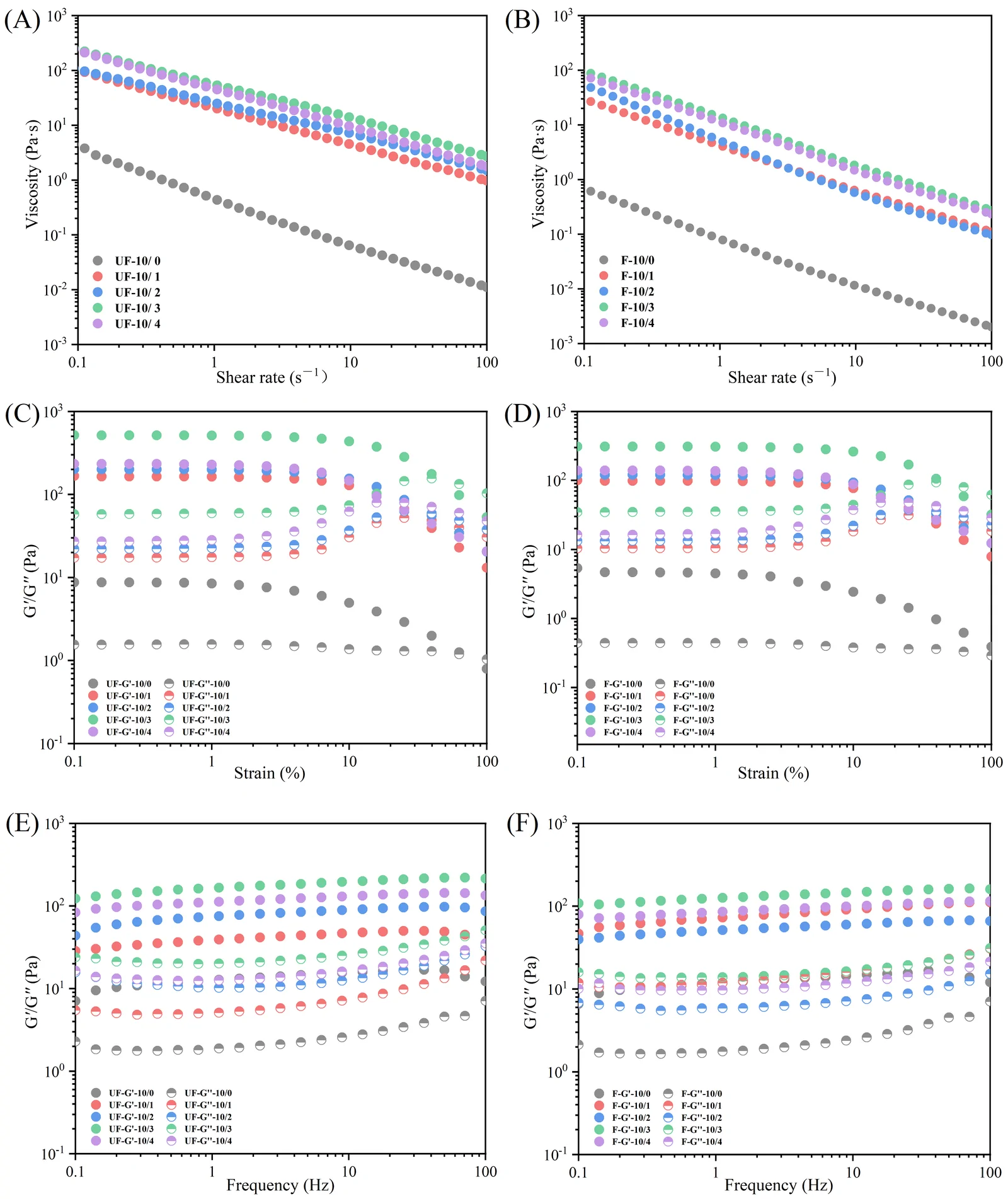
Apparent viscosity curves (**A**,**B**), strain sweep curves (**C**,**D**), and frequency sweep curves (**E**,**F**) of unfermented (UF) and fermented (F) RP/CMC-HIPEs after heating at 90 °C for 60 min.

Figure 7 presents the steady-state rheological behavior and dynamic viscoelastic properties of RP/CMC-stabilized HIPEs after heating at 90 °C for 60 min. As shown in Figure 7A,B, all samples retained shear-thinning behavior after heating. Their apparent viscosities increased overall compared with those of the corresponding unheated samples, suggesting increased resistance to flow after thermal treatment. This change may be associated with heat-induced alterations in protein conformation and interactions within the droplet structure [37]. Across the tested formulations, the CMC-containing samples continued to exhibit higher apparent viscosity than the RP-only samples, consistent with the trend observed before heating. The fermented samples also retained lower apparent viscosity than the corresponding unfermented samples, indicating that the fermentation-associated differences in flow resistance persisted after thermal treatment [38].

The strain sweep results further confirmed the structural stability of the heated HIPEs (Figure 7C,D). All samples exhibited storage modulus (G′) values higher than loss modulus (G″) values within the low-strain region, indicating that the systems retained an elastic-dominant gel-like structure after heating. The 10/0 sample, particularly the fermented 10/0 sample, showed a pronounced decrease in G′ at higher strains, suggesting relatively poor deformation resistance in systems stabilized solely by RP. In contrast, the RP/CMC composite systems, especially those with RP/CMC mass ratios of 10/2–10/4, maintained G′ > G″ over a broader strain range, suggesting that CMC-containing systems exhibited greater resistance to deformation over a broader strain range. This observation is consistent with the macroscopic appearance results, where no obvious oil release or structural collapse was observed in the RP/CMC composite systems after heating.

The frequency sweep results further showed that all heated samples maintained G′ values higher than G″ throughout the tested frequency range (Figure 7E,F), confirming that the thermally treated HIPEs retained typical weak-gel characteristics [39]. Compared with the unheated samples (Figure 3E,F), the RP/CMC composite systems exhibited higher G′ values after heating, indicating that thermal treatment strengthened the elastic network structure of the HIPEs. This change may be associated with heat-induced changes in protein conformation and interactions within the droplet network [40].

### 3.7. CLSM Images and Particle Size Analysis of Heated RP/CMC-HIPEs

**Figure 8:**
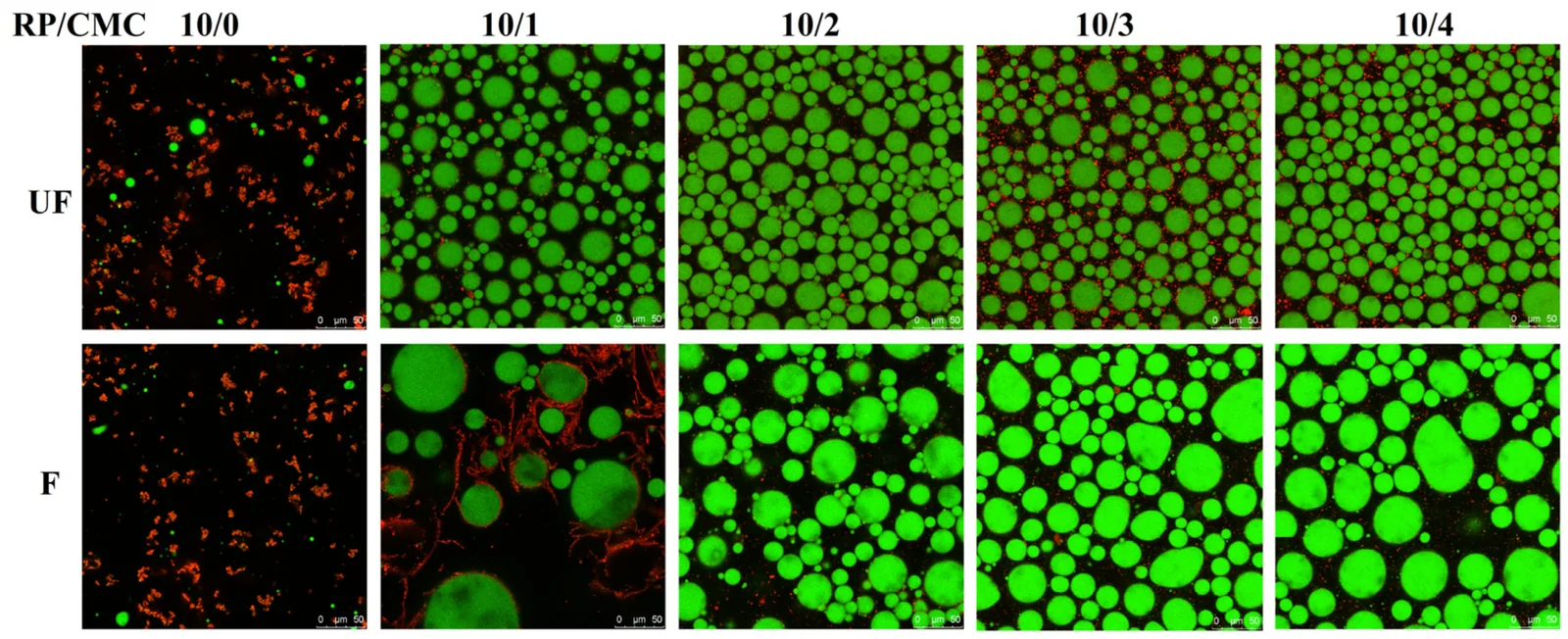
CLSM images of unfermented (UF) and fermented (F) RP/CMC-HIPEs after heating at 90 °C for 60 min.

Figure 8 shows the CLSM images of RP/CMC-stabilized HIPEs after heating at 90 °C for 60 min. After thermal treatment, a large number of close-packed green oil droplets were still observed in the CMC-containing samples, indicating that RP/CMC-HIPEs retained the characteristic microstructure of high-internal-phase emulsions after heating, without obvious droplet aggregation or oil release. This result is consistent with the semi-solid appearance and O/W dispersion characteristics observed after heating (Figure 6), suggesting that the protein–polysaccharide network may constructed with the participation of CMC maintained the microstructural stability of HIPEs during thermal treatment. Previous studies have shown that HIPEs stabilized by polysaccharide-based structures or protein–polysaccharide complexes can maintain good gel network stability under thermal treatment, and that their thermal stability is closely associated with the interfacial protective layer, continuous-phase network, and oil droplet immobilization ability [40,41].

Compared with the CLSM images before heating (Figure 4), the 10/0 sample still exhibited a relatively heterogeneous droplet distribution after heating, with obvious local droplet aggregation. This suggests that although heat-induced structural changes and aggregation of RP may have occurred, they were insufficient to promote a more uniform droplet arrangement in the RP-only system. In contrast, the RP/CMC composite systems retained a relatively intact droplet-packing network after heating. In addition, the unfermented and fermented samples showed different changes in droplet size after thermal treatment. The apparent droplet size distribution of the unfermented samples generally became smaller and more concentrated, whereas the fermented samples showed a broader droplet size distribution and an increased proportion of large droplets. This difference may be related to the distinct structural and aggregation states of rice protein before heating.

**Figure 9:**
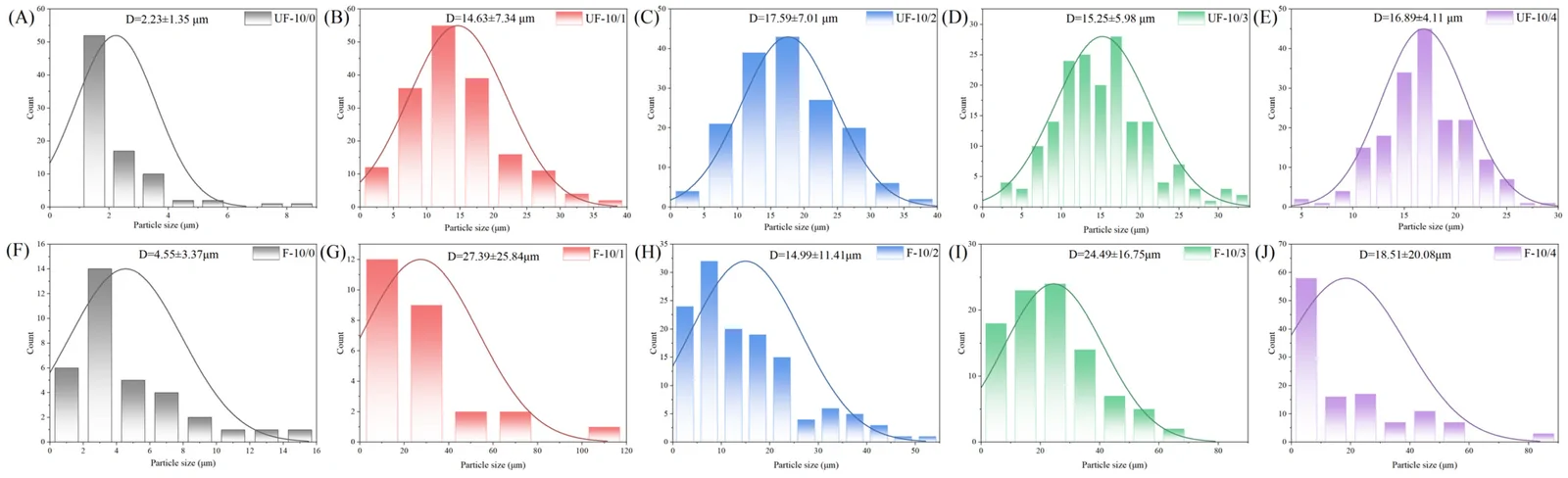
Particle size distribution of unfermented (UF) and fermented (F) RP/CMC-HIPEs after heating at 90 °C for 60 min: (**A**–**E**) are the samples before fermentation, and (**F**–**J**) are the samples after fermentation.

In the unfermented systems, rice protein had a relatively lower initial aggregation degree. Heat-induced partial unfolding and structural rearrangement of rice protein could expose more hydrophobic groups and interfacially active sites, thereby promoting the adsorption and association of RP/CMC complexes at the oil-water interface [42]. Such changes may promote the interfacial association of RP/CMC complexes and contribute to a more effective restriction of droplet movement and close approach, potentially accounting for the narrower apparent droplet-size distribution after heating. In contrast, fermentation-induced acidification and conformational rearrangement may have caused RP/CMC complexes to exist in a partially pre-aggregated state before heating [18]. Subsequent thermal treatment further strengthened protein–protein and inter-droplet attractive interactions, promoting droplet flocculation and possibly limited local coalescence, which led to an increased number of large droplets (Figure 9). Previous studies have reported that heat-induced changes in interfacially adsorbed globular proteins can enhance attractive interactions between protein-coated droplets and promote flocculation [43,44].

### 3.8. Microstructure of RP/CMC-HIPEs Observed by Cryo-SEM

**Figure 10:**
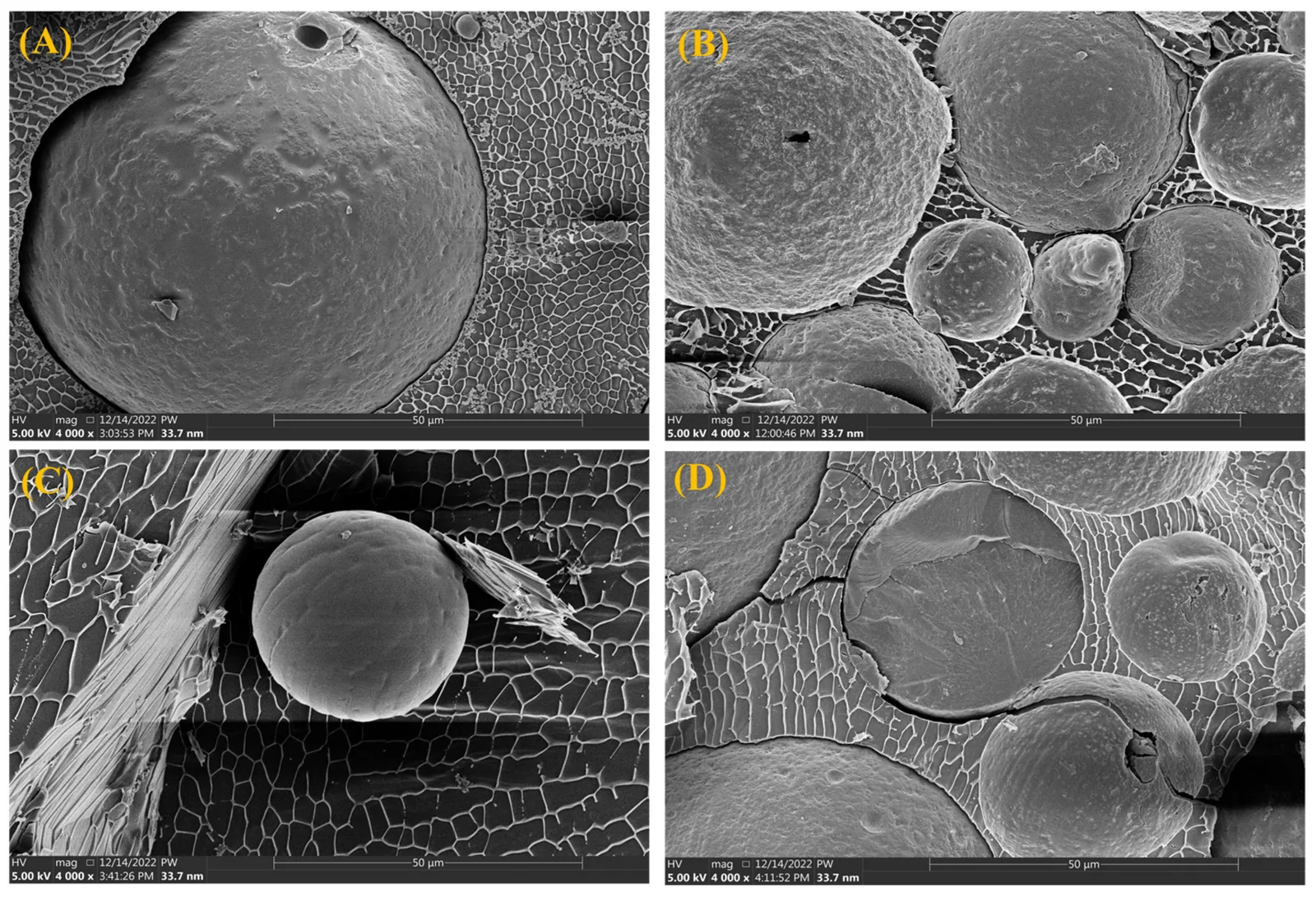
Cryo-SEM images of RP/CMC-HIPEs: (**A**) UF-10/0, (**B**) UF-10/3, (**C**) F-10/0, and (**D**) F-10/3, the magnification is 4000×.

To further elucidate the microstructural features associated with the stability of RP/CMC-HIPEs, samples with RP/CMC mass ratios of 10/0 and 10/3, both before and after fermentation, were selected for Cryo-SEM observation. As shown in Figure 10, all samples exhibited a typical close-packed droplet structure characteristic of high-internal-phase emulsions, indicating that a large number of oil droplets were immobilized within the continuous-phase network at *φ* = 0.75, thereby forming a semi-solid emulsion system. However, differences were observed in droplet organization and the surrounding matrix.

In both the UF-10/0 and F-10/0 samples, relatively large individual oil droplets were observed, and the continuous-phase network surrounding the droplets appeared comparatively loose. These features were consistent with the more pronounced phase separation observed in the RP-only samples after centrifugation (Figure 2). In contrast, the UF-10/3 sample exhibited more closely packed droplets separated by a comparatively continuous surrounding matrix. These results indicate that fermentation did not disrupt the fundamental HIPE framework of the 10/3 system. Miao et al. reported that the stability of polysaccharide-based HIPEs is closely associated with the three-dimensional polymer network formed around and between oil droplets [41]. Similarly, through CLSM and Cryo-SEM observations, Zhang et al. found that polysaccharide microgels could form a three-dimensional network surrounding oil droplets, thereby enhancing the stability of HIPEs [45].

Furthermore, comparison between UF-10/3 and F-10/3 revealed that the fermented sample exhibited smoother droplet surfaces and a relatively looser interfacial packing structure. These changes may reflect fermentation-induced alterations in the organization of the RP/CMC complexes around the droplets. Together with the lower apparent viscosity and storage modulus (G′) of the fermented samples, these observations suggest a reduction in the overall structural rigidity of the HIPEs.

To further support the structural characteristics of the optimized formulation, the physicochemical and emulsifying properties of RP/CMC complexes at the mass ratio of 10/3 were determined (Table S1), and their morphology was observed by SEM (Figure S1). The results showed that fermentation changed the morphology of the RP/CMC complexes from a smooth sheet-like structure to a more fragmented and porous form. Meanwhile, fermentation increased the free sulfhydryl group content, surface hydrophobicity, solubility, emulsifying activity index (EAI), and emulsifying stability index (ESI) of the RP/CMC complexes. The increases in free sulfhydryl group content and surface hydrophobicity suggest that protein conformation may have changed, potentially increasing the accessibility of reactive and hydrophobic groups, thereby improving the dispersibility and interfacial adsorption ability of the RP/CMC complexes. In particular, the improvements in solubility and emulsifying properties provided a physicochemical and functional basis for the formation and stabilization of RP/CMC-HIPEs.

## 4. Conclusions

This study demonstrated that RP/CMC complexes can effectively stabilize O/W HIPEs with an oil phase volume fraction of 0.75 and construct semi-solid sauce-like systems. The incorporation of CMC significantly improved the macroscopic homogeneity, self-supporting behavior, oil-binding capacity and rheological strength of RP-stabilized HIPEs. Among the tested formulations, the RP/CMC ratio of 10/3 exhibited the numerically highest oil-binding capacity, reaching 99.84 ± 0.13% and 99.69 ± 0.04% in the unfermented and fermented samples, respectively, with no significant difference from the 10/4 formulations. Fermentation did not eliminate the semi-solid appearance or gel-like rheological behavior of RP/CMC-HIPEs but reduced their apparent viscosity and storage modulus, indicating a lower flow resistance and elastic response. These changes may be beneficial for tuning the flow and deformation behavior of HIPEs intended for spreadable sauce applications. Further studies are needed to evaluate long-term storage stability and shelf life under refrigerated (4 °C) and room-temperature conditions, as well as process scale-up and production costs, to establish the feasibility of industrial application.

## Data Availability

Data available on request due to privacy restrictions.

## Supplementary Materials

The following supporting information can be downloaded at: https://www.mdpi.com/article/10.3390/foods15213995/s1, Figure S1: Scanning electron microscopy (SEM) images of RP/CMC complexes at the 10/3 mass ratio before (A) and after fermentation (B); Table S1: Functional properties of unfermented and fermented RP/CMC complexes.
