# Supplementary material for: Effect of Lactiplantibacillus plantarum Fermentation on the Structural and Functional Properties of Rice Protein/Carboxymethylcellulose Stabilized High-Internal-Phase Emulsion
Source: Foods. 2026 Nov 7;15(21):3995. doi: 10.3390/foods15213995 (PMC13762568; doi:10.3390/foods15213995)
Supplement: Supplementary file 1 [file foods-15-03995-s001.zip › foods-4609377-supplementary.pdf]

Characteristics of a High-Internal-Phase Emulsion Stabilized by a Rice Protein/Carboxymethylcellulose Complex Fermented with *Lactiplantibacillus plantarum*

Yurong Zhou [1,2], Jing Yang [1,2], Wenfei Xiong [2], Chao Chen [2], Lifeng Wang [1,2,*]

[1] College of Food Engineering, Harbin University of Commerce, Harbin 150028, China

[2] College of Food Science and Engineering, Nanjing University of Finance and Economics, Nanjing 210023, China

***Corresponding Author:**

Prof. Lifeng Wang

Tel: +86-025-86718515;

E-mail address: wanglifeng_8@163.com

Methods

The free sulfhydryl group (SH_F) content was determined using Ellman's reagent method. Briefly, 60 mg of unfermented or fermented RP/CMC complexes was dissolved in 10 mL of Tris-Glycine-8 M urea buffer and then centrifuged at 8,000 rpm for 10 min. The supernatant was collected, and 2 mL of the supernatant was mixed with 10 μL of Ellman's reagent. After vortexing, the mixture was allowed to react for 5 min, and the absorbance was measured at 412 nm. The protein concentration of the supernatant was determined using a BCA protein assay kit.

$$SH_F\ (\mu mol/g) = \frac{73.53 \times A_{412}}{C}$$

Where A_{412} is the absorbance of the sample solution at 412 nm; C is the concentration of the sample (mg/mL).

Surface hydrophobicity was determined using 8-anilino-1-naphthalenesulfonic acid (ANS) as a fluorescent probe. Unfermented and fermented RP/CMC complexes were dispersed in 10 mmol/L PBS buffer (pH 7.0) and diluted to different RP concentrations ranging from 0.01 to 0.1 mg/mL. Then, 4 mL of each sample solution was mixed with 40 μL of 8 mmol/L ANS solution, vortexed for 5 s, and kept in the dark for 10 min. The fluorescence intensity was measured using a fluorescence spectrophotometer (Hitachi F-2000, Tokyo, Japan) at an excitation wavelength of 390 nm and an emission wavelength of 470 nm. The fluorescence intensity was plotted against protein concentration, and the initial slope of the curve was used as the surface hydrophobicity index (H_0).

Solubility was determined by dispersing 100 mg of unfermented or fermented RP/CMC complexes in 10 mL of 10 mmol/L PBS buffer (pH 8.0). The dispersion was vortexed and dissolved for 2 h, followed by centrifugation at 5,000 rpm for 10 min. The supernatant was collected, and the protein content was determined by the Kjeldahl method. Solubility was calculated as follows:

$$\text{Solubility (\%)} = \frac{\text{Protein content in the supernatant}}{\text{Total protein content in the sample}} \times 100$$

The emulsifying activity index (EAI) and emulsifying stability index (ESI) were determined using a turbidimetric method. Unfermented and fermented RP/CMC complexes were dispersed in 0.01 mol/L PBS buffer (pH 8.0) to prepare sample solutions with an RP concentration of 1 mg/mL. Soybean oil was added at 25% (v/v), and the mixture was homogenized at 10,000 rpm for 1 min to form an emulsion. Immediately after homogenization, the emulsion was collected from the bottom of the tube and diluted 100-fold with 0.1% (w/v) sodium dodecyl sulfate (SDS) solution. The absorbance was measured at 500 nm. The absorbance was measured again after 10 min. EAI and ESI were calculated using the following equations:

$$\text{EAI (m}^2\text{/g)} = \frac{2 \times 2.303 \times A_0 \times D}{C \times \varphi \times 10^4}$$

$$\text{ESI (min)} = \frac{A_0 \times \Delta t}{A_0 - A_{10}}$$

where A_0 and A_{10} are the absorbance values measured at 0 and 10 min, respectively; D is the dilution factor; C is the protein concentration before emulsification (g/mL); φ is the oil volume fraction; and Δt represents the measurement time interval.

Results

Table S1 Functional properties of unfermented and fermented RP/CMC complexes

	SH_F (μmol/L)	H_0	Solubility (%)	EAI (m^2/g)	ESI (min)
UF-RP/CMC	16.80±0.26[b]	11242.5±57.50[b]	27.53±0.54[b]	7.60±0.01[b]	66.38±3.93[b]
F-RP/CMC	19.06±0.53[a]	12055.5±35.50[a]	39.71±1.84[a]	10.14±0.02[a]	82.06±0.73[a]

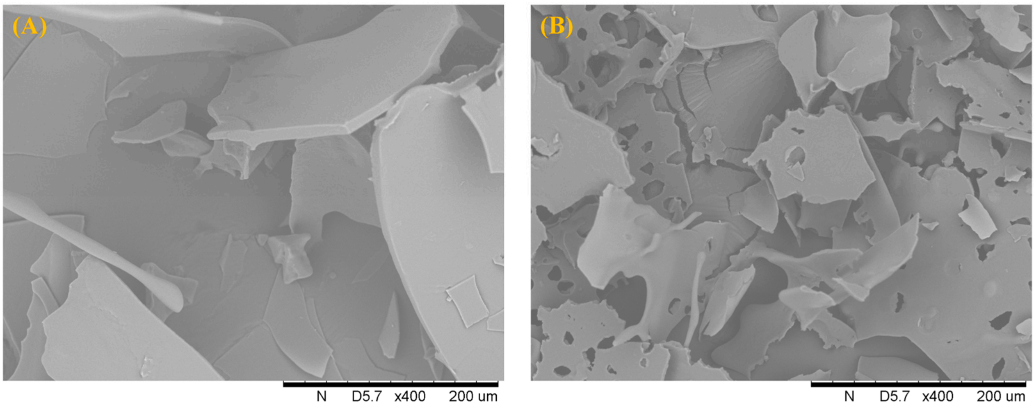

Figure S1 Scanning electron microscopy (SEM) images of RP/CMC complexes at the 10/3 mass ratio before (A) and after fermentation (B).
